# Organotin Polyethers as Biomaterials

**DOI:** 10.3390/ma2041558

**Published:** 2009-10-21

**Authors:** Charles E. Carraher, Michael R. Roner

**Affiliations:** 1 Florida Atlantic University, Department of Chemistry and Biochemistry, Boca Raton, FL 33431, USA; 2 Florida Atlantic University, Florida Center for Environmental Studies, Palm Beach Gardens, FL 33410, USA; 3 University of Texas at Arlington, Department of Biology, Arlington, TX 76019, USA; E-Mail: roner@uta.edu

**Keywords:** tin polymers, cancer, antiviral, bactericides, pancreatic cancer, organotin polyethers, polysaccharides, lignin, poly(vinyl alcohol)

## Abstract

Organotin polyethers are easily synthesized employing interfacial polymerization systems involving the reaction of hydroxyl-containing Lewis bases and organotin halides. A wide variety of organotin-containing polymeric products have been synthesized including those derived from natural and synthetic polymers such as lignin, xylan, cellulose, dextran, and poly(vinyl alcohol). Others have been synthesized employing known drug diols such as dicumarol, DES, and dienestrol and a wide variety of synthetic diols. Included in these materials are the first water soluble organotin polymers. The organotin polyethers exhibit a wide range of biological activities. Some selectively inhibit a number of unwanted bacteria, including *Staph. MRSA*, and unwanted yeasts such as *Candida albicans*. Some also inhibit a variety of viruses including those responsible for herpes infections and smallpox. Others show good inhibition of a wide variety of cancer cell lines including cell lines associated with ovarian, colon, lung, prostrate, pancreatic and breast cancer. The synthesis, structural characterization, and biological characterization of these materials is described in this review.

## 1. Introduction 

### 1.1. Organotins

The topic of organotin compounds has been recently reviewed [1]. The topic of organotin polymers has also been recently reviewed [2]. Organotin compounds are chemicals containing at least one covalent Sn-C bond and are classified as mono, di, tri and tetraorganotin compounds, depending upon the number of organic moieties on the tin atom. The first organotin compound was synthesized by Sir Edward Frankland in 1853 [1]. The use of organotin compounds has surged in the last 50 years. Tin has a larger number of its organometallic derivatives in commercial use than any other metal [1,3]. The major application (about 70%) of diorganotin derivatives is in PVC piping as a heat and light stabilizer additive. PVC is unstable under exposure to light and heat resulting in discoloration and embrittlement. In the early 1940s it was found that this degradation could be prevented by addition of certain organotin derivatives. Today, many of these stabilizers are based on organotin polymers made by Carraher and co-workers [4]. 

Organotin compounds are also utilized commercially in polymers in film for food packaging, and in PVC articles [1,3]. They are also used as industrial catalysts and as antiseptic, antifouling, and anti-mildew agents in industry and agriculture and as additives in paint formulations [1]. 

Even so, today the reason for the widespread interest in most organotin compounds emanates from their biological activity, being employed specifically as antibacterial, anti-yeast, anti-mildew and anticancer agents in industry and agriculture and as additives in paint formulations. 

Much of the recent drive towards inclusion of organotin into polymers is the result of federal legislation prohibiting use of so-called unbound or monomeric organotin compounds in paints and coatings. Trialkyltin derivatives have been used as effective biocides in agriculture and as anti-fouling agents to ward off barnacles from ship hulls and to prevent mildew and rot in commercial and residential homes [5,6,7,8,9]. While monomeric organotin compounds were widely used as antifouling and anti-mold agents they inhibited and killed nearby plant and animal life through migration of the organotin moiety [10,11,12,13]. This toxicity towards aquatic life has limited their use [10,11]. Chemically bound organotin (organotin moieties chemically a part of polymers) was permitted in the legislation. Thus, there is activity to create non-migrating chemically bound organotin materials, namely polymers, that contain organotin moieties. 

### 1.2. Organotin Polyethers 

We and others have synthesized a wide variety of organotin polyethers [14,15,16,17,18,19,20,21,22]. Our initial efforts at the synthesis of organotin polyethers in the early 1970s employed both the interfacial reaction between organotin dihalides and diols and the solution reaction between organotin dihalides and alkoxides generated from reaction of the diols with sodium. Two modified non-aqueous reaction systems were employed. In one, the organotin dihalide was dissolved in hexane and the diol along with triethylamine were added neat. Yields were generally in the range of 70-90%. In the second system, the organotin dihalide was dissolved in hexane and the diol and triethylamine were dissolved in acetronitrile. Here, yields were less, in the general range of 30-50%. After studying a number of reactions involving other Lewis bases such as diamines, salts of carboxylic acids, and mixed Lewis-base containing reactants, organotin polyethers were synthesized employing the classical interfacial system which was employed for the vast majority of subsequent syntheses [17]. While these compounds exhibit various levels of ability to inhibit microorganisms the following emphasizes bacterial, cancer and viral inhibition.

**Figure 1:**
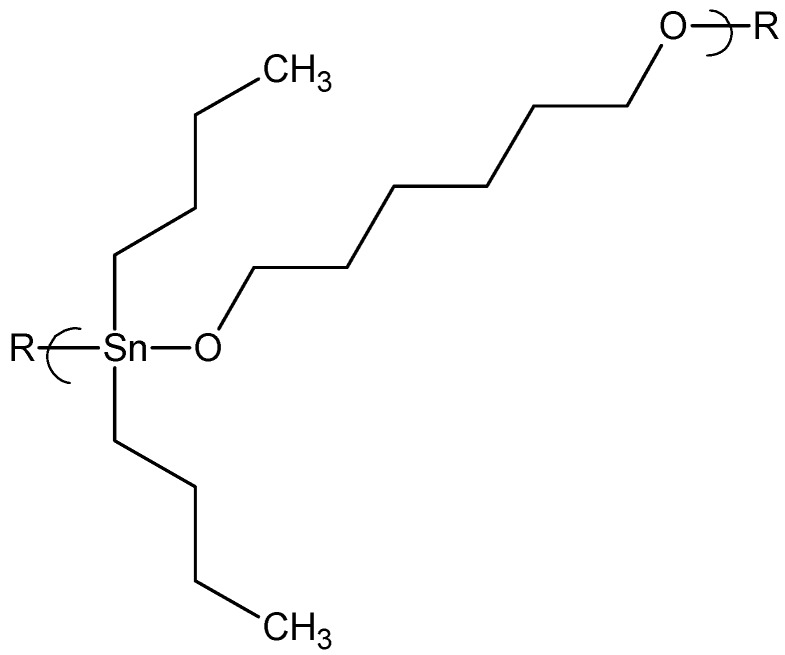
Repeat unit for the product of 1,6-hexanediol and dibutyltin dichloride.

While we had synthesized organotin polyethers in the 1970s the emphasis for the recent effort involving cancer inhibition started when we were surveying the cell growth characteristics of a number of compounds including some organotin polyethers [14,15,17]. We found that a series of organotin polyethers derived from simple aliphatic diols showed good inhibition of Balb 3T3 cells [21]. For instance, the product from dibutyltin dichloride and 1,6-hexanediol, Figure 1, showed a GI_50_ of 5 mg/mL . 

**Figure 2:**
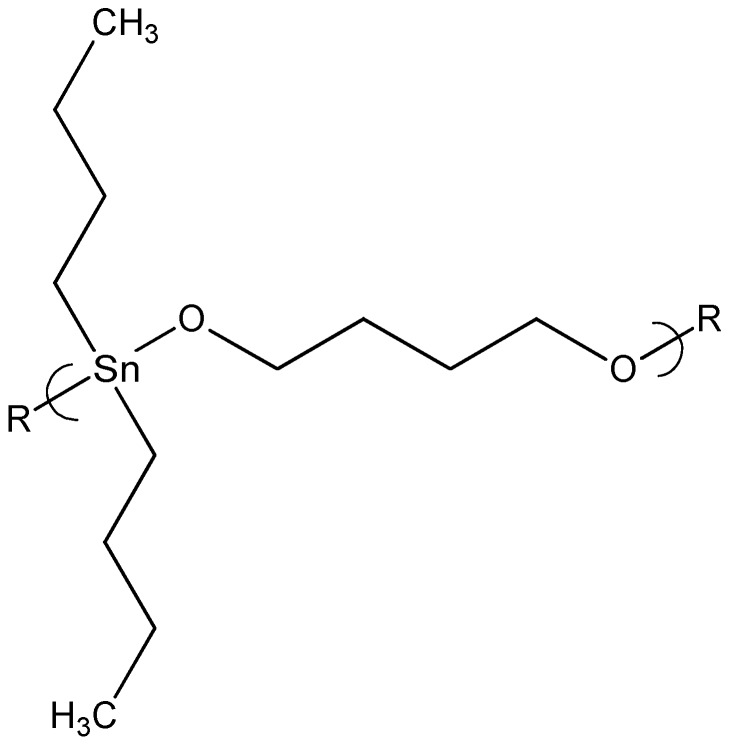
Repeat unit for the product of 1,4-butanediol and dibutyltin dichloride.

The product from 1,4-butanediol and dibutyltin dichloride (Figure 2) showed a GI_50_ of 0.25 mg/mL. 

**Figure 3:**
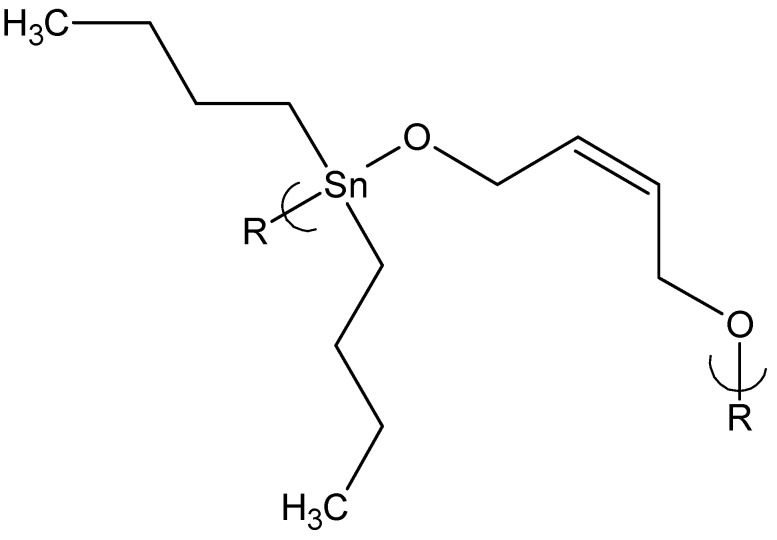
Repeat unit for the product of dibutyltin dichloride and 1,4-butenediol.

Finally, the product of dibutyltin dichloride and 1,4-butenediol (Figure 3) showed a GI_50_ value of 0.025 mg/mL, the lowest GI_50_ found to that date towards 3T3 cells of any of the tested organotin products. The GI_50_ for this 3T3 cell line for cisplatin is 0.4 mg/mL. 

These results suggested two structural windows that merited further investigation. These structural windows were, first, that activity increases as the distance between the oxygen atoms in the diols decreases. Second, that unsaturation (i.e., a pi bond), present in the diol may contribute to the ability of the organotin polyether to inhibit cell growth. 

Prior efforts indicated that organotin polymers containing the dibutyltin moiety often exhibit good inhibition in comparison to those containing other diorganotin moieties. Thus, a large study was carried out employing simple diols reacted with dibutyltin dichloride.

In addition to testing the ability of the organotin polyethers to inhibit cell growth, two other compounds were studied as benchmarks. These were the organotin monomers, and cisplatin, the most widely employed anticancer drug. 

There are different measures of the ability and effectiveness of drugs to arrest cell growth. The two most widely are employed here. The first measure of the effectiveness to inhibit cell growth is the GI_50_. This measures the concentration necessary to inhibit 50% cell growth. The second measure of effectiveness is the 50% chemotherapeutic index. The chemotherapeutic index is the concentration of the compound that inhibits the growth of the normal cells, here WI-38 cells, by 50% divided by the concentration of the compound that inhibits the growth of the cancer cells by 50%. Larger values are desired since they indicate that a larger concentration is required to inhibit the healthy cells in comparison to the cancer cells or stated in another way, larger values indicate some preference for inhibiting the cancer cells in comparison to the normal cells. 

Unless noted otherwise, the polyethers were tested against a battery of cancer cell lines including cell lines associated with human bone (143 human fibroblast bone osteosarcoma cells), lung (2RA human lung transformed fiberblast), breast (MDA MB-231 human pleural effusion breast adenocarcinoma and MCF-7 human pleural effusion breast adenocarcinoma), prostrate (PC-3 human prostate carcionoma), colon (HT-29 human recto-sigmoid colon adenocarcinoma) as well as several animal cancer cell lines (BSC-1 and vero cells Afraican green monkey kidney epithelial cells; L929 mouse transformed fibroblast cells). WI-38 cells, a human normal embryonic lung fibroblast, were used as the control representing a healthy cell line. Also, the 2RA cell line was employed. The 2RA is a WI-38 transformed cell line that is often employed to form a matched pair with the normal WI-38 human cell line. The 3T3 cells were also employed. Two pancreatic cell lines were also employed (AsPC-1 and PANC-1). These results will be described later. 

## 2. Results and Discussion

### 2.1. Biological Activities

Organotin compounds have been known for years to exert an effect on biological organisms from microorganisms to the human organism. Here we will briefly review some of these biological activities that are pertinent to the current review. 

### 2.2. General

The general microorganism biological activities of organotin compounds has been studied since the 1950s [1]. Generally, inorganic tin compounds are non-toxic or only slightly toxic towards mammals, insects, bacteria, and fungi whereas organotin compounds show varying biological activities. For alkylorganostannanes, toxicity varies depending on the alkyl group. In general an increase in the alkyl chain length gives a decrease in toxicity [1,2,23,24]. The particular pattern for methyl, ethyl, propyl, and butyl varies with test organism. For tri-*n*-alkyltin acetates, the methyl organotin compounds are the most active for insects and mammals; for fungi and certain bacteria the propyl and butyl compounds are the most active. Further, activity decreases as the number of alkyl groups decreases as follows R_4_Sn > R_3_Sn > R_2_Sn > RSn > Sn all for tin IV compounds with the anionic X group exerting little influence on activity [1,2,14,15]. The compounds most used commercially are tributyltin chloride, dibutyltin dichloride, tributyltin oxide and dibutyltin oxide since they are among the least toxic alkyltin derivatives towards people [1]. 

In almost all cases, here and within the other sections in this review, the bacterial testing for organotin polymers is done with the organotin compounds as solids. While the activities are presumably less for solids, some offer outstanding inhibitions. As solids, these organotin materials are suitable for treatment of infections (here mainly topical), contaminated sites, use as a preventative agent, and in the treatment of water sources. They can be used topically as additives to creams, cleaning detergents and soaps, coatings (paints), plastics, paper, etc. [2]. The organotin-containing drugs are rapidly (generally within 30 seconds) synthesized in good yield employing readily available reactants. Thus, ready availability on a gram to tons scale of target drugs is achievable. They can be handled without need for gloves or other protective ware and have shelf-lives in excess of several years [2]. 

Most of these organotin polymer products can be incorporated into paper, plastics, textiles, and the like with only some loss in bacterial and fungal biological activity [2]. 

Following is a listing of agents successfully inhibited by the compounds cited in the review. Agents successfully inhibited include the following: *E. coli, B. subtilis, B. catarrhalis, S. epidermidis, E. aerogenes, N. mucosa, K. pneumoniae, A. calcoacetius, A. flavus, A. niger, A. fumagatus, Penicillin sp, Trichoderma reesei, Chaetomium globosum, P. aeruginosa, S. aireis, C. albicans, T. mentagrophytes,* and *Staph MRSA* [1,2]. It must be remembered that each of these species have various strains that may or not be susceptible to inhibition by these organotin compounds.

### 2.3. Organotin Compounds and Their Anticancer Activity

Most of the efforts related to anticancer activity have involved the use of monomeric or small molecules [25,26,27,28]. Monomeric organotin compounds have been studied since 1929 as potential anticancer agents. In the 1970s and 1980s it was believed that because of the similarity of bond angles present in organotin compounds to those present in platinum compounds, namely cisplatin, that organotin compounds might act in a similar manner to cisplatin. More recent studies indicate that the activity of organotin compounds is diverse and probably is not the same as cisplatin [29]. This difference in mechanism of activity may actually be an advantage since a combining of organotin compounds with cisplatin may offer several routes to the inhibition of cancer growth. More on this latter.

While numerous organotin compounds have been investigated as potential anticancer drugs and many have shown promising activity, the structural features required for biological activity are still poorly defined. Both diorganotin and triorganotin compounds can be active [30], but the most effective groups are normally diorganotin compounds with butyl moiety and less so the phenyl moiety. The nature of the ligands in active compounds can also vary enormously. Active organotin compounds do appear to share certain characteristics, including available coordination positions on the tin and low hydrolytic cleavage of the Sn-alkyl (or Sn-aryl) bonds. There also appears to be a necessary balance between lipophilic properties needed for crossing the cell membrane and hydrophilic character required to display activity in an aqueous environment. 

The combination of two biologically active entities, however, in the same molecule has been shown in some cases to enhance their activity [31]. For example, triphenyltin(IV) derivatives of phthalic acid and salicaldehyde have significant activity toward a range of fungi [32,33]. Recently, interests in organotin(IV) carboxylates are increasing due to their possible medical uses as antitumor agents [34]. For example, the fluoro-substituted carboxylate ligands with di- and triorganotins produced several antitumor active compounds [35]. Hubert *et al*. concluded that antitumor active tin compounds possess available coordination positions around tin atom and also have relatively stable ligand-tin bonds with low hydrolytic decomposition [36]. Thioamide-organotin complexes, on the other hand, have shown high antitumor activity, which is believed related to the ligand type and not to the geometry of the compounds [37,38,39,40]. Given that the antitumor action of Sn(IV) compounds may not be due to their direct interaction with DNA constituents, their reaction with enzymes like lipoxygenase is always of interest in the attempt to elucidate their mechanism of action [37,38,39,40]. This antitumor activity of the organotin complexes follows the same order as lipoxygenase inhibition, an enzyme that is involved in the inflammation mechanism and tumor genesis [37,38,39,40].

Organotin compounds are toxic to living organisms and to the immune system [41,42,43,44,45,46,47,48,49,50,51,52,53,54]. Here we will focus on cell death. There are morphologically and biochemically two distinct forms in the mode of cell death, referred to as apoptosis and necrosis. Apoptotic cell death is morphologically characterized by cell shrinkage, the blebbing of plasma membranes, nuclear breakdown and DNA fragmentation. The biochemical hallmark of apoptosis is the cleavage of chromosomal DNA into nucleosomal units, which appears to be the final blow in the cell death process. Apoptosis is mediated by the activation of a family of cysteine proteases with specificity for aspartic acid residues, referred to as caspases. Caspases are synthesized as precursor forms, and an apoptotic signal converts the precursors to mature enzymes, which subsequently cleave other caspases that are downstream in the cascade. Caspases activated by apoptotic signals cleave various cellular substrates which may be responsible for the morphological changes that occur in the cells.

Fas ligand is expressed in activated T cells, and induces apoptosis in target cells by binding to Fas, its receptor. The Fas engagement activates a cascade of caspase proteases, as found in most apoptotic processes. Activation of cell surface receptor Fas leads to rapid inactivation of the electron transfer activity of cytochrome *c* and subsequent release of cytochrome *c* from mitochondria. In addition, the activation of cell surface death receptor leads to rapid activation of caspase-8, the apical caspase in the Fas-induced apoptotic pathway. The activation of caspase-8 initiates two pathway leading to the activation of downstream caspases. One is the activation of downstream caspases like caspase-3, caspase-6 and caspase-7 by directly cleaving them, the other is the activation of these downstream caspases indirectly by causing cytochrome *c* release from mitochondria that triggers caspase activation through Apaf-1. The release of cytochrome *c* triggers the interaction of Apaf-1 and caspase-9, which in turn results in the activation of caspase-9. Activated caspase-9 then cleaves and activates procaspase-3, an event that leads to the cleavage of other death substrates, cellular and nuclear morphological changes, and ultimately, cell death [49]. Bid, a BH3 domain-containing proapoptotic Bcl-2 family member, is a specific proximal substrate of caspase-8 during Fas-induced apoptosis that functions upstream of BclXL [50]. That is to say, Bid acts downstream of caspase-8 and upstream of BclXL in Fas-induced apoptosis. The cleavage of Bid by caspase-8 mediates the mitochondrial damage in the Fas pathway of apoptosis [51]. While full-length Bid is localized in cytosol, truncated Bid (tBid) translocates to mitochondria and thus transduces apoptotic signals from cytoplasmic membrane to mitochondria. tBid induces first the clustering of mitochondria around the nuclei and release of cytochrome *c* independent of caspase activity, and then the loss of mitochondrial membrane potential, cell shrinkage, and nuclear condensation in a caspase-dependent fashion [49]. Full-length Bid can not induce apoptosis, while tBid induces apoptosis very rapidly and efficiently. tBid-induced cell death is inhibited completely by BclXL.

Apoptosis is also accompanied by the internucleosomal degradation of chromosomal DNA. Recently, a caspaseactivated DNase (CAD) and its inhibitor (ICAD) have been identified in the cytoplasmic fraction [52]. Activation of CAD downstream of the caspase cascade is responsible for internucleosomal DNA degradation during apoptosis and ICAD works as an inhibitor of this process. In addition, of caspases, particularly caspase-3 is activated during apoptosis and cleaves inhibitor (ICAD) of CAD and inactivates its CAD-inhibitory effect [53], suggesting that ICAD could be inactivated by an apoptotic signal.

As noted before, organotin compounds are toxic to the immune system [41,42,43,44,45,46,47,48,50]. Alkyltin compounds such as dibutyltin (DBT) and tributyltin (TBT) cause severe thymus atrophy. On histopathological examination, there is marked depletion of lymphocytes and the depletion is due to cell death. The mechanism of cell death induced by DBT or TBT was investigated from the angle of apoptosis in T-lymphocytes. The results revealed that DBT induced caspase-independent cell death (necrosis), whereas TBT induced caspasede endentcell death (apoptosis). Furthermore, the following sequence is believed to occur on TBT-induced apoptosis. Bid cleavage by activation of caspase-8 produced tBid and released cytochrome *c* from mitochondria. Release of cytochrome *c* induced the activation of caspase-3 through caspase-9. CAD activated by caspase-3 induced DNA fragmentation. That is to say, it was elucidated that TBT induced caspase-dependent and mitochondria-mediated cell death.

Organotin compounds exert powerful toxic action on the immune system, brain nervous system and endocrine system. Organotin compounds such as dibutyltin (DBT), dioctyltin (DOT) and tributyltin (TBT) compounds caused immunodeficiency of cell-mediated immunity and T cell-dependent humoral immunity due to severe thymus atrophy. [41,42,43,44,45,46,47,48]. This atrophy depends on marked depletion of lymphocytes and the depletion is due to the cell death. Moreover, under long-term exposure, from five weeks to eight weeks, dibutyltin-induced atrophy is reversible, whereas tributyltin-induced atrophy is irreversible. These results suggest that there might be different mechanism between the cell death induced by dibutyltin and tributyltin [41,42,43,44,45,46,47,48].

#### 2.3.1. Mechanism of Action of Organotin Compounds

The mechanism(s) of action of tin-containing compounds is not well-understood. As already noted, there exist differences between the mechanism of action of platinum compounds and tin compounds. Tin compounds generally have a tetrahedral, trigonal bipyramidal, or octahedral structure. Platinum compounds exhibit a square planer structure. This difference in the molecular geometry might lead to the variation in binding to the cellular targets. 

Organotin compounds exhibit anti-tumor activity either effecting cell death via apoptosis or necrosis. Organotin compounds can bring about numerous cellular effects [55], including accumulation of organotin in the Golgi apparatus and endoplasmic reticulum; changes in membrane permeability and phospholipid synthesis; inhibition of DNA, RNA and protein synthesis; enhancement of cell proliferation; changes in cAMP levels and calcium mobilization; inhibition of mitochondrial and Na^+^-K^+^ ATPases and activation of enzymes associated with apoptosis [56,57,58,59,60,61,62]. Organotin compounds with demonstrated antitumor activity have been shown to bind to and unwind DNA, effect cell death by necrosis [63], and increase extracellular calcium influx and generate reactive oxygen species leading to apoptosis [59,64,65]. Thus there appears to be many cellular targets and numerous mechanisms of action for organotin compounds. There are also differences in the general toxic effects caused by tin-containing substances. Simple organotin compounds, like triethyltin chloride and dibutyltin dichloride, are known to bring about nervous system and immune system damage [66]. More complicated compounds, with good antitumor effects, that contain substituted benzoates or steroidcarboxylates showed significant toxicity in the form of paralysis and gastrointestinal disturbances [67]. However, other organotin compounds with good antitumor activity containing phenanthroline, lupinylsulfide, or carnosine showed very little general toxicity [63,68]. Therefore, a number of organotin compounds have promise as effective anticancer agents but do so with varying unwanted side effects and through different mechanisms. 

#### 2.3.2. Polymeric Drugs-Advantages

Polymers offer several potential advantages compared to small, monomeric drugs [69,70]. First, a polymer should be filtered out by the kidneys more slowly than small compounds [71] decreasing kidney damage and increasing the time the compound remains in the body to attack the cancer. Second, a large polymer may be effective against tumors that have developed resistance to other chemotherapeutic agents, either because the polymer is not recognized by cellular resistance mechanisms or because the polymer could be used to deliver local, high-dose chemotherapy [72]. Third, the size and structure of the polymer would provide more binding sites to cellular targets, increasing effectiveness. Fourth, a polymer can be designed to incorporate multiple anticancer agents within the same molecule that act against cancer cells by different mechanisms. Fifth, the polymeric structure can permit easy coupling to other molecules, such as those that specifically target cancer cells, allowing delivery of a polymeric drug to a particular site without impairing its effectiveness. Sixth, a polymer can be designed as either a large stable compound that enters the cell by pinocytosis [73,74] and is active in a polymeric form, or as an unstable compound that slowly degrades into active monomeric units in a timed-release fashion [75,76]. Seventh, large molecular weight molecules accumulate in solid tumors more than in normal tissues because of the enhanced permeability and retention effect, resulting in high amounts of polymers in the interstitial space due to a leaky vasculature and limited lymphatic drainage typical of tumors [77]. Finally, polymers can be synthesized to fine-tune many structural characteristics, such as monomeric components, chain length, cross-linking, and polarity, to maximize anticancer activity and vary the spectrum of activity. Thus, polymers have great flexibility in their design and many possible benefits compared to monomeric drugs. 

#### 2.3.3. Solubility

As noted throughout the review, cross-linked organotin materials are, as expected, insoluble. The linear materials are generally soluble in dipolar aprotic liquids such as hexamethyphosphorous triamide (HMPA), dimethyl sulfoxide (DMSO), *N,N’*-dimethylacetamide (DMA), and *N,N’*-dimethyl-formamide (DMF) [2]. 

Linear organotin polymers produced in rapidly stirred systems are often more soluble in acetone and other polar liquids if the acetone or polar solvent is added just after recovery and prior to allowing the mixture to become dry [2]. This is presumably due to the presence of entrapped solvent molecules that prevent close chain interactions causing the chains to be more easily approached and replaced by the solvent molecules. Thus, such metastable states allow the organotin polymers a monetary solubility in additional solvents.

For measuring biological activity solvent interaction with the organism to be tested is critical. Thus, initial solubility in DMSO and then addition to water is the preferred approach for testing of not only our compounds, but is the solvent and procedure of choice industry-wide [2,70]. For cell line testing we have also employed HMPA but typically employ either water alone, for the water soluble polyethers, or initial solubility in DMSO [78]. While HMPA is a better solvent for some of the polymers, it is toxic in cell lines at a lower concentration than DMSO. 

#### 2.3.4. Stability

Solid organotin polymers are stable in air and under room conditions to over several decades [2]. The organotin polymers show greater stability in acids than bases [2]. This tendency for overall greater stability in acid than in base can also serve as a means of the delivery of both the organotin and entire chain since its good acid stability should allow it “safe” passage past the digestive tract and the blood being slightly basic might encourage some degradation of the polymer chain.

#### 2.3.5. Physical Nature

Most organotin polymers are solids but a few are gummy in nature, probably because entrenched solvent has not been entirely removed. They are generally white but can become colored if the matrix in which the organotin moiety is attached is a color site, such as in the organotin polydyes where the organotin moiety is attached to commercial dyes such as fluorescene [2]. 

#### 2.3.6. Thermal Properties

Most of the organotin products show good long term room temperature stability as solids. When heated they begin to degrade above 100 °C so they can be heated to 50 °C without worry [2]. As a general rule, organotin polymers undergo degradation without melting. Weight loss generally occurs through a series of stability plateaus where there are temperature ranges, often several hundred degrees, where little or no weight loss occurs [2,79]. For reference, most commercial polymers undergo weight loss in a continual fashion once weight loss begins [80]. This is because for these polymers the overall thermal stability is governed by a single bond, the carbon-carbon bond. This is not true for most metal-containing polymers. 

Some of the organotin polymers retain over 50 % of their weight to 900 °C [2] The nature of this residue is not well understood but is believed to contain the metal in some highly complex cross-linked matrix. The generally lesser stabilities in air are believed to be due to the availability of low-lying d-orbitals on the tin that are susceptible to attack by oxygen leading to oxidative degradation of the organotin chain. 

#### 2.3.7. Mass Spectrometry

Various mass spectrometry techniques have been carried out to assist in the structural identification of organotin ethers. For reasonably high resolution mass spectrometry, the presence of tin is an advantage. Tin has ten naturally occurring isotopes, seven of which appear at abundances of several percent and greater. This allows the ready identification of the presence of tin within ion fragments. Depending on the type of mass spectrometry, we have been able to obtain decent isotopic abundance matches for ion fragment clusters containing up to five tin atoms. 

We have recently been employing MALDI MS for the identification of a number of metal and non-metal containing polymers [81,82]. The technique employed by us is not straight forward MALDI MS. Classical MALDI MS requires that the material be soluble in a suitable solvent. A “suitable solvent” means a solvent that is sufficiently volatile to allow it to be evaporated prior to the procedure. Further, such a solvent should dissolve both the polymer and the matrix material. Finally, an ideal solvent will allow a decent level of polymer solubility, preferably a solubility of several percentage and greater. For most synthetic polymers, these qualifications are only approximately attainted. Thus, traditional MALDI MS has not achieved its possible position as a general use modern characterization tool for synthetic polymers. By comparison, MALDI MS is extremely useful for many biopolymers where the polymers are soluble in water. Even so, we have employed a variety of MALDI MS that appears to be suitable for poorly or insoluble samples. Even though MALDI MS is a mild or gentle technique causing a minimum of fragmentation, fragmentation is observed in polymer samples by us and others [81,82].

We have employed MALDI MS to assist in the structural identification of organotin polymers including organotin polyethers [2,81,82]. In general, it is found that bond scission preferably occurs at heteroatomed sites along the polymer backbone producing ion fragments. The interpretation of spectra is not straight forward. At masses below 1,000 Da there are a number of ion fragment clusters that contain organotin, but organotin that has reacted with the matrix [83]. We have employed a number of matrixes including 2,5-dihydroxybenzoic acid and α-cyano-4-hydroxycinnamic acid. It is not surprising that products are formed since these matrices contain acid and alcohol functional groups that are known to react with tin. Thus, caution is to be exercised when interrupting the spectra. Even so, MALDI MS has proven to be a powerful tool in the identification of the repeat unit of the organotin polyethers, with ion fragment clusters containing five units identifiable using isotopic abundance analysis and higher units identified by simply looking at the evolution of various ion fragment clusters. 

### 2.4. Biological Activities of Polysaccharides

The microorganism activity of these polymeric products has been investigated. The results for the saccharide and polysaccharide-derived products has been discussed in the introductory portion of this review and has been reviewed elsewhere [2]. The organotin polysaccharide-products have not been tested for anticancer or antiviral activity mainly because of an often lack of suitable solubility of the materials. Eventually, it should be possible to vary the triorganotin content to the extent that water soluble products are formed. 

One question concerns the source of inhibition. Are the organostannane-modified polysaccharides toxic themselves or are the microorganisms simply not able to digest them and die due to starvation? In general, the plates containing the microorganisms contain nutrient agar such that there should be little question with respect to starvation. Even so, studies were carried out using two microorganisms, *T. reesei* and *C. globosum*, that are known to digest polysaccharides such as cellulose and dextrose [84,85]. For one set of experiments, dextrose was added to test tubes containing dibutyltin-modified cellulose. The second set of tubes was identical except no dextrose was added. The growth decrease for both sets of tubes was similar. This is consistent with the butyltin-containing cellulose being toxic to the microorganisms and not simply having the modified cellulose material inhibiting or preventing saccharide metabolism. 

Not unexpectedly, the biological activities of the organotin-containing products from sucrose are similar to those from the complex carbohydrates since the primary driving moiety for biological activity is the organotin moiety [2,86]. 

With the appearance of new resistant strains of common bacteria the need for new treatment rationales increases. As part of our efforts we have looked at particularly dangerous microorganisms where the “cure” is either extreme or not available. One of the most modern day insidious microorganisms involved in nosocomial infections is methicillin-resistant *S. aureus*, MRSA (also called *Staph MRSA*). This microbe commonly colonizes those patients who are seriously ill and in high-risk areas such as intensive care and burn units. It is also a significant risk factor in surgical wound infections. The incident of MRSA infection in hospitals is increasing at an alarming rate. It can be carried in the anterior nares of otherwise healthy health-care givers and transferred to the patient during routine attendance at the patient’s bedside. Other areas suspected of harboring the organism are air handling duct work, linens, and general room contamination. It has now become part of the public sector. We developed a number of organotin-containing polymeric materials that inhibit MRSA and which can be incorporated into soaps, cleaning agents, coatings, etc. for the purposes of prevention and decontamination [87]. These include products derived from dextran, cellulose, PVA and lignin. 

The ability of these organotin-containing polymers to successfully inhibit a wide number of microorganisms, including such resistant bacteria as MRSA, makes them prime candidates in the war on biological terrorisms for the treatment of such microorganisms as *Bacillus anthracis* responsible for anthrax, *Yersinia bub*. responsible for bubonic plague, *Francisella tularensis* responsible for tuleramia, etc. Some of these products are also active against a number of fungus that cause mildew and rot and against the microorganisms responsible for ringworm and athletics’ foot [88]. 

In general, these materials can offer longer "shelf life", sustained release of the active agent, greater retention of the active agent due to lowered solubility of the polymer-all of these resulting in a greater effect of the controlled release formulation with less active agent in the environment.

### 2.5. General Properties and Cancer Inhibition of Organotin Polyethers

The first group of studies involved investigating the notion that the ability to inhibit cancer cells was related to the distance between the oxygen atoms. In the initial study polymers were formed from diols such as diethylene glycol and tetraethylene glycol as well as hydroxyl-capped polyethylene glycol, PEG, compounds [22]. Since the diols can be considered a series of ethylene oxide units containing hydroxyl ends the group is referred to as ethylene oxide derived polymers or EG-derived polymers. We also used hydroxyl-terminated poly(ethylene glycols), PEGs, which we will use the term polymeric PEG to differentiate them from the monomeric EGs such as pentaethylene glycol. Here, the distance between the oxygen atoms was an ethylene unit. The n values were 1-5 and for the hydroxyl-terminated poly(ethylene glycols) they were 9 to 225. The PEG derived polymers are water soluble and represent the first instance of water soluble organotin polymers. 

The polymeric PEG diols are described in terms of the designation PEG and a number which is the molecular weight of the particular polymeric PEG. Thus, PEG8,000 or PEG8000 has (8,000 Da/44 Da/repeat unit) about 180 ethylene oxide repeat units separating the two terminal hydroxyl groups. 

The products were polymeric with degrees of polymerizations, DPs, in the range of 300 to 2,000 for the non-PEG diols. The PEG products from PG8,000 and PG10,000 were oligometic with DPs of about 20. Further, yields were in the range of 35 to 100 % except for the high molecular weight PEGs where the yields were about 5%. It is believed that the low yields for the higher molecular weight poly(ethylene glycols), PEGs, may be due to their high solubility in the aqueous phase. Since the product is recovered as a precipitate in a water-containing reaction system, the water solubility of the PEG product will result in a loss of product yield. An alternate explanation is related to the difficulty of the dibutyltin dichloride or dibutyltin monochloride end-group finding an unreacted hydroxyl because of the simple large distance between end groups in the relatively large poly(ethylene glycol) diols resulting in a lowered product yield. This may also account for the low DPs for these products.

**Figure 4:**
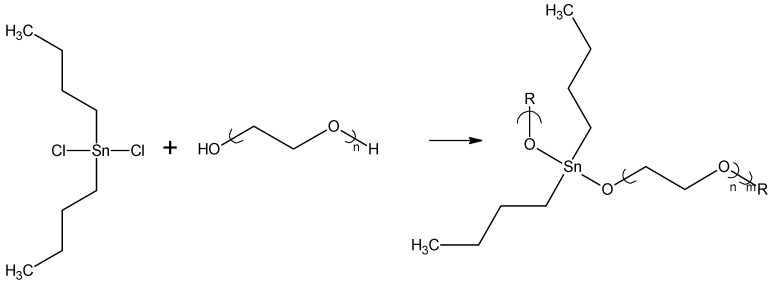
Reaction between PEG and dibutyltin dichloride.

We were also able to vary the chain length of the EG and PEG products though use of non-molar amounts of mono-hydroxyl-containing alcohols [89]. Thus, for the PEG400 product the molecular weight was decreased from 76,000 Da to 41,000 Da when the aqueous phase contained an equal molar amount of the PEG400 and 1-butanol. 1-Butanol is a monofunctional alcohol that will stop chain growth when added to a growing chain since it has no alcohol group remaining once it has reacted with one dibutyltin dichloride. 

The stability of the polymers in DMSO and water (for the water-soluble products) was studied over a period of over two years [22,90]. The polymers are stable for several months with a molecular weight half life, the time for the chain length to be halved, on the order of 60 to 80 weeks for all of the polymers. Further, the stability of the water soluble polymers in water and DMSO is approximately the same.

We also examined the enzymatic degradation of the water soluble polymers [91]. There exist hundreds of enzymes that are present in our bodies that are responsible for metabolic degradation. We chose trypsin because it is readily available and its activity is well established. Trypsin is a serine protease found in our digestive system where it breaks down proteins. It predominately cleaves amide linkages at the carboxyl moiety of lysine and arginine unless either is followed by proline. It is employed in a number of biotechnological processes. Our cell inhibition studies generally take three days. For one experiment with the polymer derived from PEG8,000 the polymer was stable over the test period of four days in both water alone and in the presence of trypsin. This is consistent with a lack of marked enzymatic degradation by trypsin over this period of time. 

The polymers were examined for their ability to inhibit cell growth. There did not appear to be a definite trend with respect to the number of ethylene oxide units separating the tin atoms. In comparison with cisplatin, the non-PEG samples showed values in the same range as cisplatin. By comparison the PEG products, with significantly lower fractions of organotin moiety present, give higher GI_50_ values with the PEG8,000 and MW10,000 samples in DMSO giving values generally higher than for the other tested samples. At very low amounts of organotin moiety GI_50_ values appear to be significantly greater consistent with the importance of having a sufficient amount of the organotin moiety present to promote good cell inhibition. 

As noted before, the PEG derived polymers were water soluble and represents the initial report of water soluble organotin polymers. This allowed several studies to be carried out that ordinarily could not be carried out. We studied the activity of the PEG-derived polymers both initially dissolved in water and initially dissolved in DMSO. For the MW400 sample, modestly lower GI_50_ values are generally found for the entire water sample but for the MW8000 and MW10,000 samples there appears to be no clear trend with both the DMSO and the water samples giving similar higher GI_50_ values in comparison to the non-PEG polymers. Thus, there does not appear to be a major difference whether the polymer is originally dissolved in DMSO or water. This study is continuing.

In general, CI_50_ values of 2 and larger are considered significant. Larger values are desired since they indicate that a larger concentration is required to inhibit the healthy cells in comparison to the cancer cells or stated in another way, larger values indicate some preference for inhibiting the cancer cells in preference to the normal cells. There are a number of CI_50_ values of equal to and greater than 2. The general trend is that polymers with higher tin contents have a greater number of incidences of CI_50_ values equal to and greater than 2. There are some exceptions such as PEG(8000 and 10,000) that have generally high GI_50_ values for the cancer cell lines but relatively low GI_50_ values for the WI-38 healthy cells resulting in some of the highest CI_50_ values. Of special interest are CI_50_ values of 2 and greater for the PC-3 cell line which is reported to be the most resistant and hardest to inhibit of the available prostrate cell lines. Also of note is the large number of values equal to and greater than 2 for the head-to-head comparison between the WI-38 cell line and the 2RA transformed WI-38 cell line consistent with the majority of the organotin samples preferentially inhibiting the transformed cell line in comparison to the healthy cell line. 

There is also the question of the influence that the organotin moiety is present in a polymer rather than active as a monomer. This is measured by comparing the CI_50_ values normalized against the CI_50_ value for dibutyltin dichloride. Here, large values indicate a favorable behavior because of the presence of the dibutyltin moiety in a polymer. In almost all cases, this ratio is larger than two consistent with there being a positive influence of the organotin moiety included as part of the polymeric drug. 

The CI_50_ and GI_50_ values appear to be generally independent of the diol for the ethylene glycol through the pentaethylene glycol (n = 1-5). This is consistent with the important factor being the Sn-O linkage and not with the distance between the dibutyltin moieties.

**Figure 5:**
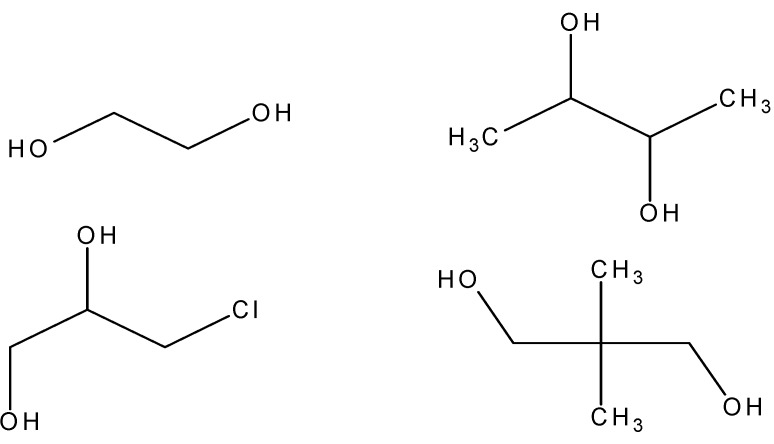
Structure of (from left to right) ethylene glycol (top), 2,3-butanediol (top), 3-chloro-1,2-propanediol (botton), and neopentyl glycol (bottom).

The second part of this subsection deals with polyethers synthesized from diols where the distance from between the oxygen atoms and the tin moiety a single ethylene or propylene unit [92]. These diols can be considered derivatives of ethylene and propylene glycol and as such results for the analogous ethylene glycol polymer are also included for discussion. For easy identification, the structures of the four diols used in this part of the study are given in Figure 5.

The GI_50_ values for the healthy cell line, WI-38, are lower for cisplatin in comparison to these dibutyltin polyethers. But the GI_50_ values for the all of the polymers, except that derived from ethylene glycol, are much lower for the cancer cell lines with some among the lowest thus far found for 3T3 and MCF-7. The MDA cell line results are consistently low for the polyethers. Thus, while the non-ethylene glycol derived polyethers generally show good inhibition of all cell lines, the polyethers show differentiation with respect to which cells are inhibited the most. The generally good inhibition by the non-ethylene glycol derived polyethers of the PC-3 cell lines is significant since, as noted before, this is known as the most aggressive of the prostrate cell lines. 

All of the polyethers show a number of CI_50_ values greater than two with some showing much greater values. The only cell line without values greater than two is the HT-29 colon cell line. The CI_50_ and GI_50_ values are both consistent with the short-chained polymers being good candidates for further study as effective anticancer drugs.

**Figure 6:**
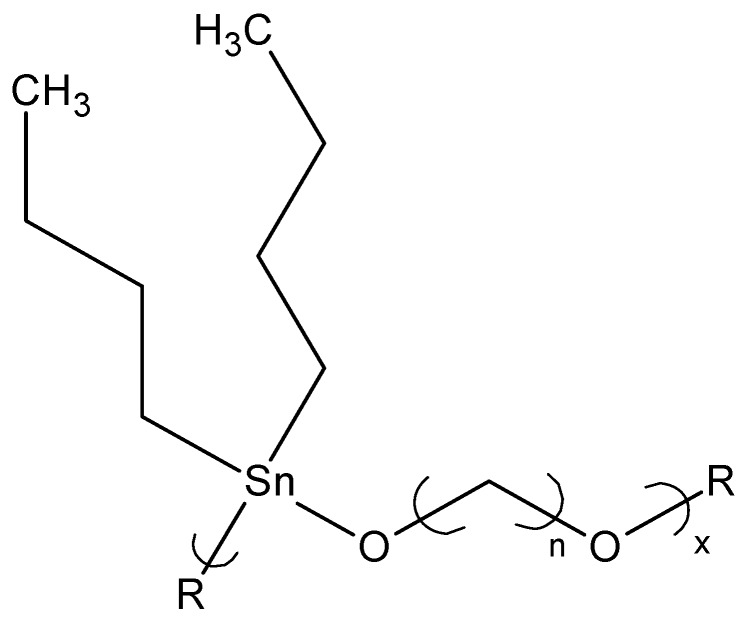
Repeat unit for the product of various methylene diols and dibutyltin dichloride.

A second series was studied where the distance between the oxygen atoms is varied by the number of methylene units between the alcohol end-groups in the diols [93,94]. Here n = 1–8 (Figure 6). Since the differentiating factor is the number of methylene units separating the alcohol groups, this series is referred to as the methylene series. Solubility of the diol in water is the limiting factor with respect to the length of the diol that could be used. Thus, the 1,8-octanediol was only water soluble with heating so was the longest diol employed with 1,9-nonanediol not soluble even with heating. 

For the lower number of methylene units separating the diols, product yield is good (about 80–90%). But as the number of methylene units increases, product yield decreases (40–50%). This may be due to space related requirements such that the incidence of collisions having the required geometry to produce the desired product is lowered for these diols. The DP ranges from 11 to 600. 

The GI_50_ values for the polymers are generally in the same range as found for cisplatin [94]. The results vary somewhat with respect to the tumor type. For instance, for HT29 and MCF-7, the polymers exhibited overall good inhibitions, often at a 20 fold lower concentration in comparison to cisplatin. There appears to be no consistent trend with respect to the number of methylene units separating the oxygen atoms. For instance, for the PC-3 prostrate cell line GI_50_ values are the highest for the ethylene glycol and 1,6-hexanediol products and the lowest GI_50_ values for the 1,4-butanediol and 1,8-octanediol products. For the MDA polymers, the lowest values are found for the 1,5-pentanediol and the 1,7-heptanediol and 1,8-octanediol products. Thus, the relationship between structure and ability to arrest cell growth is complex for this series with no clear general structural trends with respect to GI_50_ values. 

The CI_50_ values are generally less than 2, and are in fact generally less than 1. Further, the CI_50_ values for cisplatin are also small, at times smaller than those for the polymers. Thus, the CI_50_ values are indicative that the drugs preferentially inhibit healthy cells instead of the cancer cells. There does not appear to be any marked decrease in inhibition as the distance between the Sn-O units increases. This is not consistent with the notion that decreasing distance between the Sn-O units is a positive structural window of activity. 

**Figure 7:**
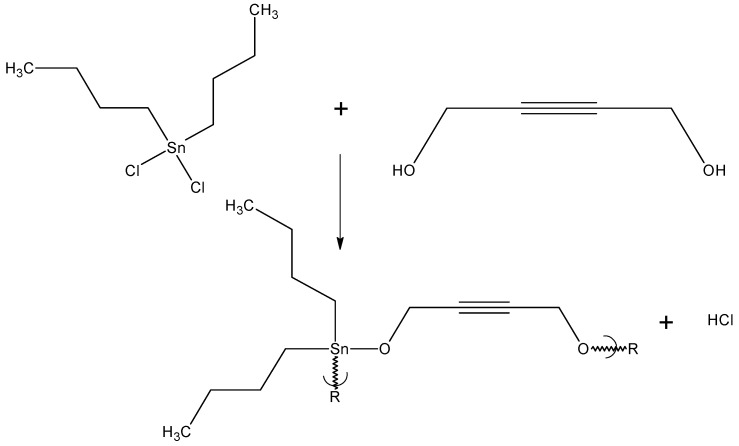
Reaction between dibutyltin dichloride and 2-butyne-1,4-diol.

The next group of experiments looked at the effect of the presence of unsaturation [95,96,97]. The first two polymers contained diol moieties containing triple bonds. Polymers from 2-butyne-1,4-diol (Figure 7) [95] and 2,5-dimethyl-3-hexyne-2,5-diol [96,97] were synthesized and their ability to inhibit cancer cell growth was found to be generally superior to those from the aliphatic-derived diols that contained no unsaturation. The GI_50_ values were generally lower and the CI_50_ values generally greater. This is consistent with the presence of unsaturation having a positive effect on the products ability to inhibit cancer cell growth. 

Both polymers also formed fibers as they were synthesized [95,97]. This behavior has been found for many metal-containing polymers and is believed to be due to the stiffness of the polymer [98,99]. This formation of fibers is referred to as anomalous fiber formation not because the fibers are unusual, but rather because they are formed in an unusual manner. Most fibers are formed by introduction of the polymer melt forced through a spinneret or into a nonsolvent or they are formed directly from the melt by pulling a small portion of the melt material resulting in fiber production. In the case of anomalous fiber formation, the fiber is formed as it is collected from the reaction system. For our syntheses, the product is formed as a precipitate and is recovered using a Buchner filter with suction. The product is collected on filter paper and then washed onto a glass petri dish using acetone. At this point, the polymer is only partially, if at all, soluble in the acetone. In some cases the fibers are present on evaporation of the liquid. More typically, as is the case here, fiber is formed as the polymer is scrapped using a flat-ended steel spatula as it is collected to add to the specimen bottle. It appears that the mechanical agitation is sufficient to induce fiber formation. It is known that the interfacial polymerization system has an orienting effect on forming polymers because of the polymerization occurring near a two-dimensional interface. This orienting affect may assist in the formation of these fibers [98,99].

The fibers derived from 2-butyne-1,4-diol are generally clear and colorless. They are smooth and linear with some having small branches that come off the main fiber. Some of the fibers are 1 mm in length and 0.007 mm diameter corresponding to aspect ratios (length to diameter) generally greater than 100. Using needle points, the fibers can be bent without breaking. The non-fiber portion is present as somewhat flat plates. 

**Figure 8:**
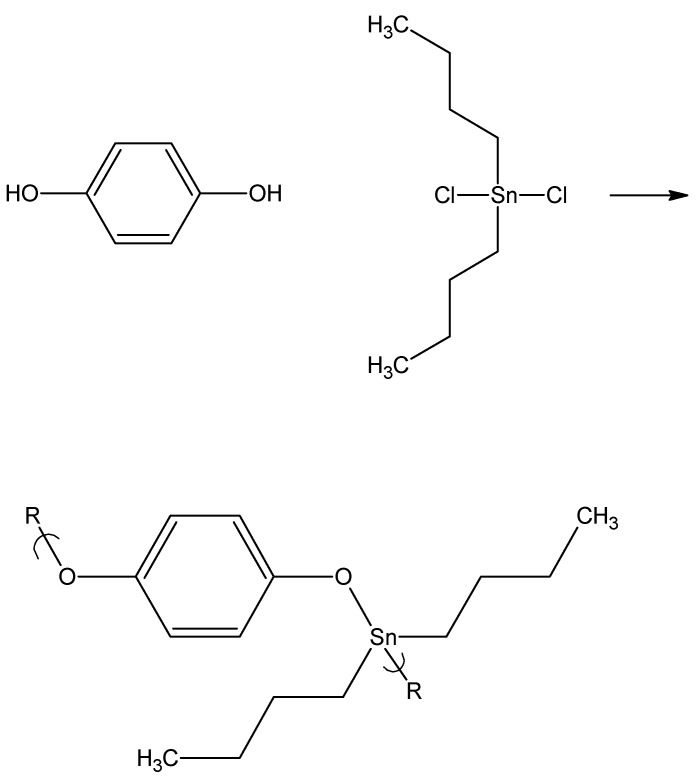
Reaction of dibutyltin dichloride with hydroquinone.

The next series of compounds also were intended to evaluate the effect of the presence of aromatic unsatuation on the ability to inhibit cancer cell lines [100]. This series consisted of a number of dibutyltin products formed from the reaction of hydroquinone and hydroquinone derivatives, Figure 8.

There were several reasons for this study. First, to test if organotin polymers derived from aromatic diols offered enhanced ability to inhibit cancer. Second, to evaluate the relationship between electronic effect on the hydroquinones and their ability to inhibit cancer cell growth. Third, to evaluate if there is a relationship between the streic nature of the diol and the ability to inhibit cancer cell growth. In general order of decreasing electron density on the hydroquinone moiety the following diols were studied: methoxyhydroquinone, *t*-butylhydroquinone, 2,5-di-*t*-butylhydroquinone, methylhydro-quinone, phenylhydroquinone, hydroquinone, 2,3-dicyanohydroquinone, bromohydroquinone, chloro-hydroquinone, 2,5-dichlorohydroquinone, tetrachlorohydroquinone, and 2,5-dihydroxybenzaldehyde. 

The GI_50_ values for the polymers are similar to those of cisplatin with some noticeable differences. First, the polymers are generally less toxic, that is greater GI_50_ values, than cisplatin for the WI-38 healthy cell line with GI_50_ values between 5 to over 50 times that of cisplatin for the cancer cell lines. The polymers exhibit low GI_50_ values particularly for the MDA breast cancer cell lines with some values approaching 100 times lower than those for cisplatin. These MDA GI_50_ values are some of the lowest found by us in our study with other organotin compounds. There does not appear to be a strong trend with respect to the electron density on the hydroquinone. 

The series di-*tert*-butylhydroquinone, *tert*-butylhydroquinone, methylhydroquinone, and hydro-quinone form a series with decreasing steric requirements. Again, there does not appear to be a strong general trend with respect to steric requirements on the hydroquinone moiety and GI_50_ value. 

About 60% of the CI_50_ were over 2 with some of them over 10. Thus, the polymers show good preference to inhibit cancer cells in comparison to the healthy WI-38 cells. But there are differences. The results for the PC-3 prostrate cells are significant. All of the electron rich hydroquinone-derived polymers exhibit good inhibition of the PC-3 prostrate cancer cells relative to the other compounds. Further, the only comparison that does not have a significant number of values over 2 are those with the breast cell line, MCF-7. The breast cancer cell line without estrogen (MDA MB-231) showed better test results than the breast cancer cell line that is positive for estrogen (MCF-7) perhaps because some of the drug is bound to the estrogen receptors and not available to act within the cell. This result is similar to that found for other organotin compounds such as those from DES as the diol. Since DES is a synthetic estrogen this may be a reasonable explanation but none of the hydroquinone compounds are employed as synthetic estrogens but the presence of the phenyl-O may be sufficient to have the hydroquinone derivatives act like estrogen mimics in being tied up by the MCF-7 cells.

Almost all of the polymers showed values of the ratio of the CI_50_ values normalized against the CI_50_ value for dibutyltin dichloride larger than one and most greater than two. This is again consistent with a favorable behavior because of the presence of the dibutyltin moiety in a polymer. 

Thus, while the GI_50_ values are in the general range as for the aliphatic-derived polyethers, the CI_50_ values are superior consistent with the favorable presence of the aromatic-derived diols. 

One of our ploys in prior studies is to include Lewis bases that themselves are biologically active. Here it is hoped that a synergic effect occurs and that the two moieties will affect the target at two different sites making it less probable for the intended target to ward off the drug. Following are two such studies. In both series, the diol was a synthetic hormone and the nature of the organotin was varied. 

**Figure 9:**
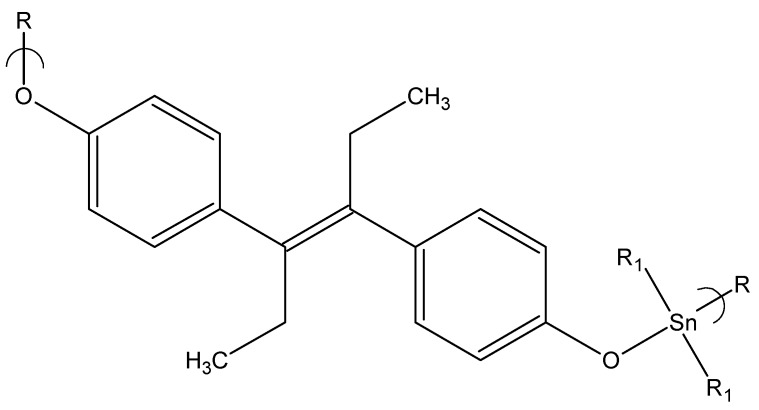
Repeat unit for the product of dibutyltin dichloride and diethylstilbestrol.

The first of these studies involved diethylstilbestrol, DES, as the diol (Figure 9) [101]. The products are generally produced in reasonable yield (about 70%) and with DPs from 20 to 200. The diol offers several differences in comparison to some of the other diols studied. Analogous to the hydroquinone derivatives, the diol contains aromatic units or sites of unsaturation. Second, unlike all of the other diols (except for dienestrol discussed shortly) employed in the present studies, DES itself offers a decent ability to inhibit the cancer cell lines studied. 

The GI_50_ values for the polymers are generally in the same range and lower than for cisplatin, Bu_2_SnCl_2_, and DES. Unlike most other studies when the nature of the alkyltin unit is varied, there is no clear trend with respect to cell inhibition and nature of the alkyl unit on tin. With the exception of the WI-38 cells, there is a relationship between general inhibition for the polymers and DES itself but not between the polymers and dibutyltin dichloride itself. Further, the breast cancer cell line without estrogen (MDA MB-231) showed better test results than the breast cancer cell line that is positive for estrogen (MCF-7) perhaps because some of the drug is bound to the estrogen receptors and not available to act within the cell. This is consistent with the finding that DES is effective against estrogen receptor positive (ER+) tumors [102,103].

Several features are apparent. First, there is a difference in CI_50_ values between the WI-38 and 3T3 cells. CI_50_ values for the WI-38 are almost all greater than one with the majority of them being greater than two (75%), the generally accepted threshold for there being a significant difference in cell inhibition between the cancer cell lines and the healthy WI-38 cell line. For the 3T3 cell line comparison values are generally less than one (92%) consistent with a tendency of the compounds to favor inhibition of the 3T3 cells in comparison to the cancer cells. One difference between the WI-38 and 3T3 cells is that the WI-38 are human in origin whereas the 3T3 cells are mouse. Additionally the WI-38 cells are truly normal, are mortal and are very sensitive to contact inhibition. The 3T3 cells are somewhere down the path to being transformed as they are no longer mortal but immortal, but are still sensitive to contact inhibition. Second, as in the case with the GI_50_ values, there is a correlation between the CI_50_ values for DES and the polymer results but not the CI_50_ values for Bu_2_SnCl_2_. Thus, the primary “driving force” for distinguishing cell inhibition appears to be the DES in the present study. Third, the nature of the organotin moiety appears to be a secondary factor in determining the ability to inhibit the cells. The most effective polymers contain the dibutyltin moiety followed closely by the diphenyltin and dipropyltin moiety but this trend is not dominant.

Again, a comparison between the polymer and dibutyltin dichloride GI_50_ values was made. The division between values related to the WI-38 and 3T3 cells continues. For the WI-38 most of the values (71%) are greater than one but for the 3T3 cells most values are less than one (67%). For both the WI-38 and 3T3 cell results values often deviate greatly from one consistent with the units being part of a polymer affecting their ability to inhibit cell growth. If the influence was simply related to the ratio of each reactant within the polymer then values should be within the realm of 0.5 to 2 because the organotin and DES moieties have approximately the same mass and same frequency factor with each chain unit having one organotin and one DES-derived moiety. 

A similar study was undertaken except employing another known steroid, here dienestrol [104,105]. The products are produced in reasonable yield (60–80%) and are polymeric with degrees of polymerization (average number of repeat units per chain), DPs, generally over 1,000. The GI_50_ values for the polymer are generally in the same range and lower than cisplatin and Bu_2_SnCl_2_. They are in the same range as dienestrol consistent with dienestrol having the capacity to inhibit cell growth. The GI_50_ values towards the MDA cells are particularly noteworthy with cell inhibition for the diethyltin and dibutyltin polymers in the order of nanograms/mL. The breast cancer cell line without estrogen receptors (MDA MB-231) showed better test results than the breast cancer cell line that is positive for estrogen receptors (MCF-7) perhaps because some of the drug is bound to the estrogen receptors and not available to act within the cell. This is similar to that found for the hydroquinone and DES-derived organotin polymers and is consistent with the finding that DES is effective against estrogen receptor positive (ER+) tumors [102,106]. 

**Figure 10:**
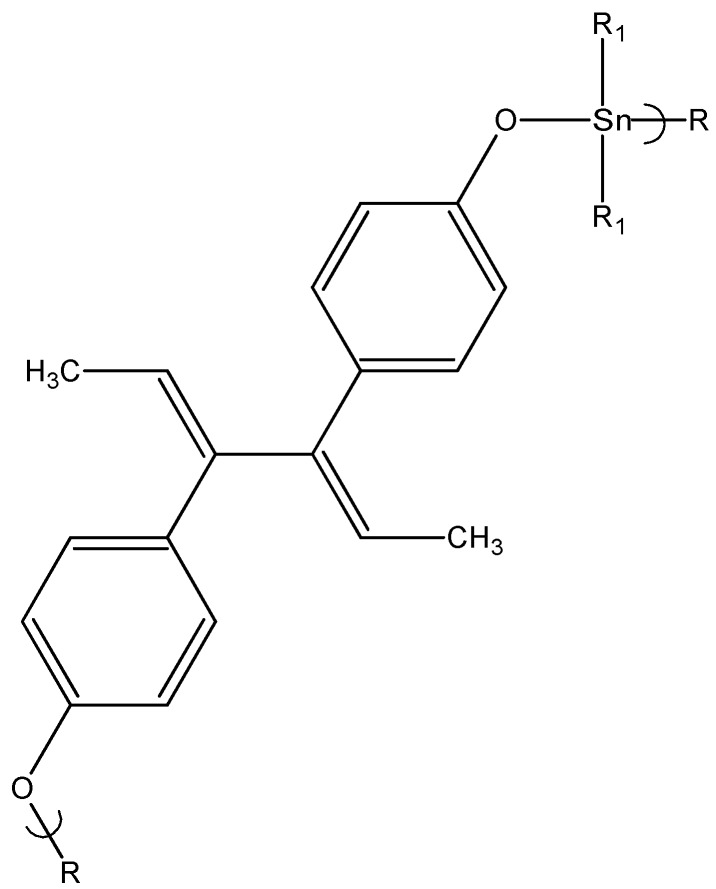
Repeat unit from the reaction between organotin dihalides and dienestrol.

While the dibutyltin polymer generally gave the lower GI_50_ values, this differentiation between the nature of the organotin moiety is not as large as with most other studies. The results are similar to the DES-organotin polymers where while the dibutyltin polymer generally gave the lowest GI_50_, cell inhibition was also closely related to the ability of DES itself to inhibit cell growth. Not unexpectedly, the studies related to both of the synthetic hormones, DES and dienestrol, are similar and both of the hormones show decent inhibition of the cell lines.

The most active compounds based on the CI_50_ values are the methyl and ethyl followed by propyl and butyl. This is not the typically found result and is believed, as in the case of DES, to be due to the ability of dienestrol itself to inhibit cell growth. The highest CI_50_ values are found for the 3T3 and MDA cells for dienestrol itself which coincides with the values found for the organotin polymers. 

Pancreatic cancer afflicts close to 32,000 individuals each year in the United States and 168,000 worldwide, and nearly all patients die from the ravages of their disease within 3 to 6 months after detection. It is the fourth leading cause of cancer death worldwide behind lung (1.3 million deaths/year), stomach (1 million deaths/year), and liver (660,000 deaths/year). Treatment of pancreatic cancer is rarely successful as this disease typically metastasizes prior to detection. Current therapies consist of surgery and, possibly, radiation and chemotherapy. Standard chemotherapy for patients with locally contained cancer includes gemcitabine. Gemcitabine has been demonstrated to improve the quality of life through better pain control, adequate performance status, decreased analgesic consumption, shrinkage of tumor, and prolonged survival. Radiation therapy is usually ineffective except as an adjunct to chemotherapy or as a palliative measure. There is no chemotherapy for metastasized pancreatic cancer.

We tested about 100 products for their ability to inhibit two pancreatic cell lines [107]. The cell lines tested are AsPC-1 which is an adenocarcinoma pancreatic cell line and PANC-1 which is an epithelioid carcinoma pancreatic cell line. Both are human cell lines and are widely employed in testing for inhibition of pancreatic cancer. Included in these tests were organotin polyethers that offered the best GI_50_ and CI_50_ values. It was found that only the products from PEG showed good CI_50_ values, generally greater than 10, some with values nearing 60. Thus, these materials are scheduled to undergo live animal testing.

**Scheme 1:**
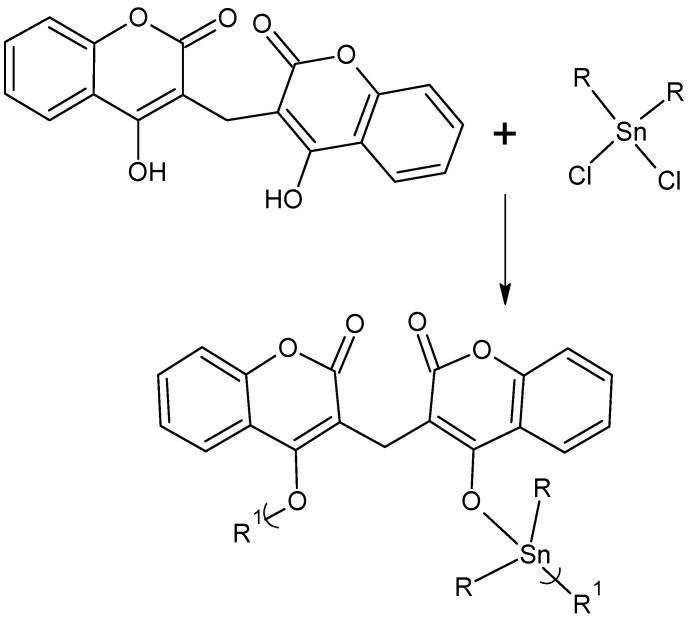
Reaction between organotin dihalides and dicumarol.

More recently, we tested polyethers derived from the anticoagulant dicumarol (Scheme 1) [108]. These materials also showed good CI_50_ values, between 7 and 8, against these two pancreatic cancer cell lines. Thus, as we synthesize additional products we are finding more polymers that successfully inhibit these pancreatic cell lines. 

#### 2.5.1. Summary of Cancer Results

In summary, it appears that the idea that short distances between the oxygen atoms contributes to better inhibition of cancer cells is not well established and depends on the particular diol. The second idea that the presence of unsatuation contributes to better inhibition of cancer cells appears to be better established. Thus, future studies might focus on compounds that contain unsaturation in the diol. Further, the organotin polyethers generally offer broad spectra inhibition of the cell lines with specific compounds offering some differentiation between the particular cancer cell lines most effectively inhibited. 

#### 2.5.2. Bacterial

The organotin polyethers were tested for their ability to inhibit a variety of bacteria and yeast including prokaryote gram-positive bacteria [*Staphylococcus aureus*, *Bacillus subtilis* (several strains)] and gram-negative bacteria (*Klebisiella aerogenes, Pseudomonas aeroginosa, Alcligenes faecalis* and several strains of *Escherichia coli*) and eukaryot (*Caldida albicans*, *Saccharomyces cerevisiae*) yeasts [109]. With the exception of the water soluble PEG products, they were tested as solids [110]. Many exhibit good inhibition of a range of microorganisms [100,109,110,111]. The water soluble PEG products inhibit all of the tested organisms. Thus, the other organotin polyethers would also probably inhibit many of the organisms if they were initially dissolved in a solvent such as DMSO or HMPA. In such a case, the toxicity of these solvents towards the tested organisms would first have to be established. Following are results for solid test samples. 

With the exception of the soluble PEG polymers, none of the EG products exhibited good inhibition of the test organisms [110]. The same is true for the methylene series except that the 1,5-pendanediol and 1,4-butanediol products showed moderate inhibition of a number of the bacteria including both gram positive and negative bacteria [110]. 

For the hydroquinone series, there is no activity for the hydroquinone products containing electron donating groups but there is some activity for the hydroquinone materials that contain electron withdrawing groups [100,112]. Thus, the polyethers from bromohydrqouinone, dichlorohydroquinone, and tetrachlorohydroquinone all show decent activity against a variety of both gram positive and gram negative bacteria. None of the products showed inhibition of either of the eukaryote organisms. It is not known why the products derived from hydroquinones possessing electron withdrawing groups are more active. It is possible that the presence of such electron withdrawing groups increase the susceptibility to some sort of metabolism-associated hydrolysis mechanism releasing the organotin moiety resulting in the observed increase in inhibition. 

The dienestrol products showed variable inhibition [111]. All showed some inhibition of the gram positive bacteria. The dibutyltin product exhibited good inhibition of both gram negative and gram positive bacteria. Most also showed some inhibition of *C. albicans*. Dienestrol itself exhibits no inhibition of any of the test organisms. By comparison, for the DES products the order of inhibition has the dimethyltin and diethyltin products inhibiting almost all of the microorganisms with the dipropyltin and dibutyltin showing lesser inhibition [109]. 

In summary, the nature of which organotin-diol combinations will offer good inhibition of the microorganisms is complex. Even so, some exhibit good inhibition of most of the test organisms and might be suitable for use as solid additives to coatings, adhesives, caulks, sealants, paper and other materials that need protection from such microorganisms. 

#### 2.5.3. Viruses

We have just begun the testing of the organotin polyethers for their ability to inhibit viruses [100,113,114,115]. The initial viruses focused on are Vaccinia and Herpes Simplex viruses. Both are DNA viruses and must be tested separately because they respond to antiviral agents differently. The HSV-1 virus is the Herpes Simplex virus which is responsible for at least 45 million infections in the US, or one out of five adolescents and adults. The Vaccinia virus is the vaccine strain for smallpox. Vaccinia strains are often considered as one of the viruses that may be used in germ warfare. 

Testing involves several steps. First, is a simple screening to evaluate which samples might be decent antiviral agents. A number of the organotin polyethers demonstrate some ability to inhibit the two viruses [100,113,114,115]. These include the dibutyltin products with ethylene glycol, pentaethylene glycol diol, 1,6-hexanediol, 1,3-propanediol, 1,7-heptanediol, the water-soluble PEG polymers, and a wide variety of hydroquinone derivatives including *tert*-butylhydroquinone, 2,5-di-*tert*-butylhydro-quinone, methylhydroquinone, phenylhydroquinone, hydroquinone, 2,3-dicyanohydroquinone, chlorohydroquinone. In fact essentially all of the substituted hydroquinones except those containing the most electron-withdrawing substituents showed some ability to inhibit the growth of at least one of the viruses. 

The second study measured the ability of the drug to inhibit cells that have been infected by the particular virus. Those that were able to effectively inhibit viruses growth in cells infected by the HSV-1 virus were dibutyltin polymers derived from the following diols: ethylene glycol, PEG400, 1,7-heptanediol, *tert*-butylhydroquinone, 2,5-di-*tert*-butylhydroquinone, phenylhydroquinone, and 2,3-dicyanohydroquinone. Acyclovir, a common antiviral agent, was used as a control. The polymers from *tert*-butylhydroquinone, phenylhydroquinone, and 2,3-dicyanohydroquinone showed inhibition values similar to acyclovir. 

Those showing the ability to inhibit viral growth in cells infected by the Vaccinia virus were derived from dibutyltin and the following diols: PEG10,000, 1,4-butanediol, 1,7-heptanediol, *tert*-butylhydroquinone, 2,5-di-*tert*-butylhydroquinone, and phenylhydroquinone. Acyclovir offered no protection against the Vaccinia virus. 

While the studies are limited, they suggest that some of the organotin polyethers show good inhibition of both viruses. Further testing is underway. 

## 3. Experimental

### 3.1. Synthesis of Organotins

The organotin-containing polymers described here were synthesized employing the interfacial polycondensation process. The interfacial process was popularized by Morgan and Carraher in the 1960s and 1970s and is today employed in the industrial synthesis of polycarbonates and aromatic polyamides known commercially as aramids [116,117,118,119,120]. This process requires what is referred to as high energy reactants, namely reactants that allow reaction to occur at somewhat low activation energies, generally in the range of 10–20 Kcal/mole (40 to 80 KJ/mole). For polyether synthesis, the hydroxyl-containing reactant is dissolved in one solvent, typically water, along with an added base such as sodium hydroxide. The organotin halide-containing reactant is dissolved in a largely water immiscible liquid such as hexane. The two phases are brought together with rapid stirring and the polymer is typically formed within less than a minute. 

There are several interfacial systems that have been employed in the production of organometallic polymers. Briefly the three most used systems are:

1. The classical system where the Lewis base, generally along with an added base, is dissolved in water and the second phase consists of the Lewis acid, here the organotin-containing reactant, dissolved in a suitable organic liquid.
2. The non-aqueous two-phase systems employ two liquids that are largely immiscible in one another such as dissolving the Lewis base in acetonitrile, nitrobenzene, or 2,5-hexadione and the acid chloride, here the organotin reactant, in a non-polar organic liquid such as hexane, decane, or carbon tetrachloride.
3. The non-organic solvent systems where the Lewis base is dissolved in water and a liquid Lewis acid such as tripropyltin chloride is used neat.

By far the most widely used interfacial system is the classical aqueous system. Other modifications to the interfacial systems have been employed to assist the reaction such as salting out and the use of phase transfer agents. As the name implies, interfacial systems require that there be two largely immiscible liquids each containing one of the reactants. Reaction occurs at or near the interface. Reactions between the organotin halides and diol-containing Lewis bases are believed to occur within the organic phase. We studied the kinetics of a related system except employing silanes in place of the stannanes [121]. Here the importance of having the reactants at the surface of the droplets is reinforced. 

The reactants are not equally distributed throughout a solution. Since “like-likes-like-the-best” there is a molecular-level segregation of the solute and solvent. As the solute to surface ratio decreases there comes a point where the surface is preferentially occupied by the solute, here the reactants. This situation occurs for interfacial systems, both unstirred and highly stirred where the surfaces of the two different liquid droplets, the interface, is especially high in the reactants. Thus, as the Lewis base meets the surface droplet holding the organotin halide it sees at the surface a greater than expected amount of organotin halide. This may be a major reason why polymer formation can occur instead of hydrolysis because on a molar basis the number of water molecules that can hydrolyze the organotin halide severely outnumber the Lewis bases within the entire phase, but not necessarily at the interface surface. (For a reaction employing for one phase 5 mmole of reactant dissolved in 50 mL of water, the ratio of water molecules to reactant molecules is 600 to 1.)

This leads us to a brief discussion of hydrolysis rates. While organic acid chlorides hydrolyze rapidly when introduced to water, organotin dihalides are relatively stable in water unless wetted. Thus, dibutyltin dichloride can be placed in boiling water for some time without noticeable hydrolysis occurring [2]. 

As noted before, the interfacial polymerization requires so-called high energy reactants such as organic acid chlorides. Although the organotin polymer groups are named as though the organotin moiety is similar to an alkylene, organotin halides are more similar to acid chlorides in their reactions and activation energies with respect to reactions involving traditional Lewis bases. Thus, the activation energies for the reactions of organotin halides with alcohols, and salts of acids is believed to be on the order of 40 to 80 KJ/mole, similar to the analogous reactions of organic acid halides with alcohols [2]. Thus, polymers are formed though a chain-wise kinetic process rather than the step-wise process. 

The classical rope trick has as the polymer forming step the pulling of the nylon rope from the system [122]. Polymerization is rapid and in rapidly stirred systems (as generally used) the polymer is formed almost instantaneously and completed within several seconds to minutes. Stirred systems act to increase the interfacial area and consequently the reaction rate. The use of simple emulsifying jars as the reaction vessel allows the interfacial surface to be increased hundreds of thousands of times when employing rapid stirring. In general, interfacial surface increases with increasing stirring rate and up to some limit so does reaction rate. There comes a limit where the amount of reactant to coat the droplets is too small to accomplish this objective. At this point increased stirring rate does not increase reaction rate. We generally use rapid stirring in the range of 18,000 rpm no load. This is several times the speed of a model airplane propeller and is easily accomplished by most commercial blenders. Because of the possibility of spark formation and subsequent fire, we employ blender systems that resist this. We have run thousands of reactions and have never had a spark fire due to the malfunctioning of the blender system. This stirring rate is about 70% the speed where large molecules are sheared. 

**Scheme 2:**
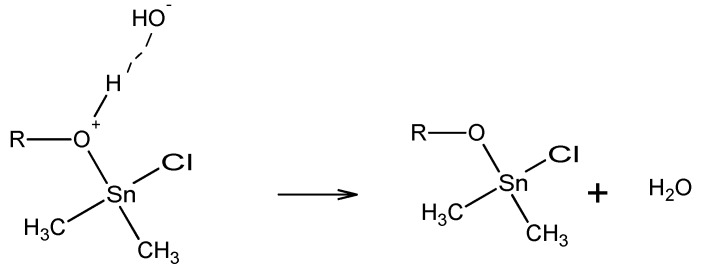
Description of the ability of added base to assist in reaction.

Added base is normally employed to convert generated acid into a salt and to prevent formation of inactive non-Lewis bases. The added base such as sodium hydroxide is also believed to assist the removal of the proton on the alcohol making it appear more nucleophilic (Scheme 2). 

Many of the products are base unstable and acid stable. In cases where added base is required there is often a race between polymer formation and base-induced degradation. Base degradation is stopped once the base is neutralized. This occurs by simple addition of dilute acid, such as dilute hydrochloric acid, after several seconds to minutes. Base degradation can also be limited through the use of various base systems where the pH is maintained at a low level. This can be accomplished though the use of only slightly soluble bases such as calcium hydroxide and barium hydroxide. It can also be lowered using organic bases as trimethylamine. Sterically hindered bases are often used since the amine competes with the desired Lewis base reactant. The use of any base will be competitive with the desired reaction and results in chain termination and the presence of the associated base as an end group. Thus, organotin reactions employing triethylamine generally have amine end groups that are readily identified through the use of infrared spectroscopy and looking at bands in the 2,900 to 2,700 cm^-1^ region derived from the triethylamine moiety. The pH control can also be accomplished through the use of buffer systems such as combinations of phosphate salts. The precipitation from the reaction solution probably also assists in protecting especially base sensitive products allowing them to be recovered.

This base instability and acid stability is useful in medical applications where medications given by mouth are initially subjected to stomach acid and if delivery of the drug is to be after the stomach, then acid stability is desirable. There are certain places in the body which are mildly basic and if the delivery site is there then the stability pattern is doubly advantageous. 

Most reactions are carried out at room temperature minimizing undesired migration and rearrangement reactions. This is particularly important when employing certain stereo-specific drugs. 

High concentrations of reactants and poor solvents are often employed since there is an advantage to employing reactants that are not highly soluble in the particular phase so that the tendency to coat the interface and move into the other phase is encouraged. There is often an upper maximum where higher concentrations actually cause markedly lower product yields. Thus, while relatively high concentrations are often desired, this must be moderated and experimented with to see what the best concentration level is for the particular reaction system. For many of the organotin systems we employ about 3 to 5 mmole in 30 to 50 mL liquid for each phase (ca 0.5 Molar).

There may also be a downside to employing poor solvents as the organic liquid. Such systems also cause rapid precipitation of the product and thus may limit molecular weight. We believe that this may be one cause for the limited chain lengths generally observed [2]. We used to use such organic liquids as carbon tetrachloride and chloroform since both of these liquids are generally poor solvents for the organotin halides employed. These liquids are no longer environmentally acceptable especially for large scale preparations. We have moved towards the use of alkane liquids such as hexane, heptane and octane which offer only moderately poor solubility for the organotin reactants but are inexpensive, readily available, and readily recyclable. A downside is that they are somewhat volatile and can ignite. 

#### 3.1.1. Preformed Polymers

In this section we will look at organotin ethers derived from reaction of the hydroxyl-containing reactant contained on a polymer with organotin mono and dihalides. In essence, any polymer that contains alcohol groups can be reacted with organotin halides. This includes most members of the polysaccharides including glycogen, starch, and cellulose. 

Migdal and co-workers were one of the first to chemically bond organotin moieties to existing polymers [2,123,124,125,126]. Polyesters and polyethers with attached organotin groups were prepared by treating these polymers with an organotin oxide, hydroxide, halide, or acetate. They also looked at naturally derived materials with the appropriate functional groups including methyl cellulose, starch, hydroxyethyl cellulose, nitrocellulose, and cellulose acetate. Some of these materials exhibited inhibition of selected microorganisms [2,123,124,125,126]. 

Industrially a re-emergence of the use of natural polymers as feedstocks and materials in many old and new areas is occurring. Since natural polymers are typically regeneratable or renewable resources, nature continues to synthesize them as they are harvested. Many natural polymers are available in large quantities. Of interest here, cellulose composes about one-third of the bulk of the entire vegetable kingdom, being present in corn stocks, tree leaves, grass, and so on. With the realization that we must conserve and regulate our chemical resources comes the awareness that we must find substitutes for resources that are not self-renewing, thus, the reason for the increased emphasis towards the use and modification of natural, renewable polymers.

Natural feed stocks must serve many human purposes. Carbohydrates as raw materials are valuable due to their actual or potential value. For example, industrial plants producing protein are utilizing rapidly reproducing re-engineered bacteria that metabolize cellulose wastes converting it to more protein-rich bacteria that is harvested and then used as a protein source feed-meal for animals. Further, natural materials can be used themselves in applications now reserved largely for only synthetic polymers. There is available sufficient natural materials to supply both food and polymer needs.

Here we will describe the synthesis of organotin-containing materials mostly based on natural hydroxyl-containing materials. 

#### 3.1.2. Lignin

**Figure 11:**
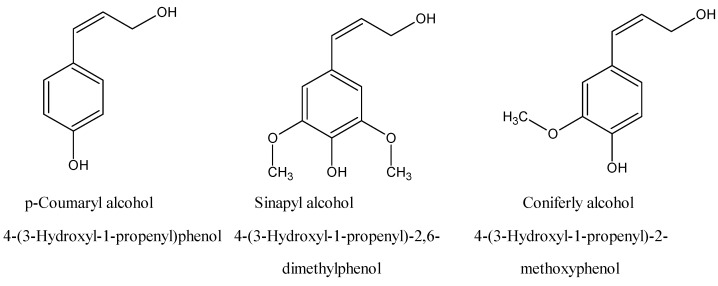
Major component structures in lignin.

Lignin is the second most abundant natural, renewable material after cellulose. It is produced at an annual rate of about 2 × 10^10^ tons and is present in the biosphere at a level of about 3 × 10^11^ tons. Based on pulp and paper production, lignin is produced worldwide from woody plants in mills at an annual rate of 5 × 10^7^ tons. As in the case with most natural polymers, the particular structure of both the original polymer and resulting product obtained from reaction with organostannane halides is variable. Lignin is a complex variable structure containing a mixture of three monolignol units methoxylated to different amounts. These monolignol units are *p*-coumaryl alcohol, sinapyl alcohol, and coniferyl alcohol (Figure 11). 

**Figure 12:**
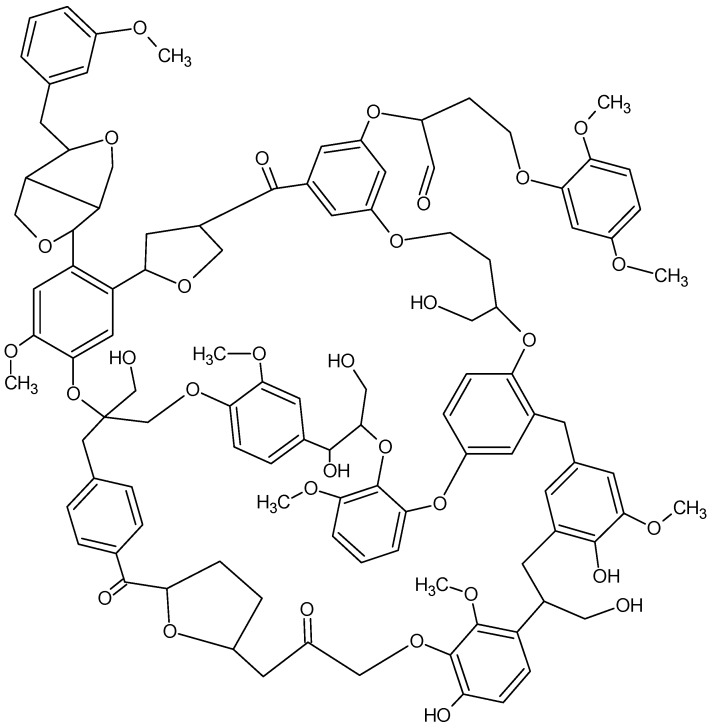
Representative structure of lignin.

**Figure 13:**
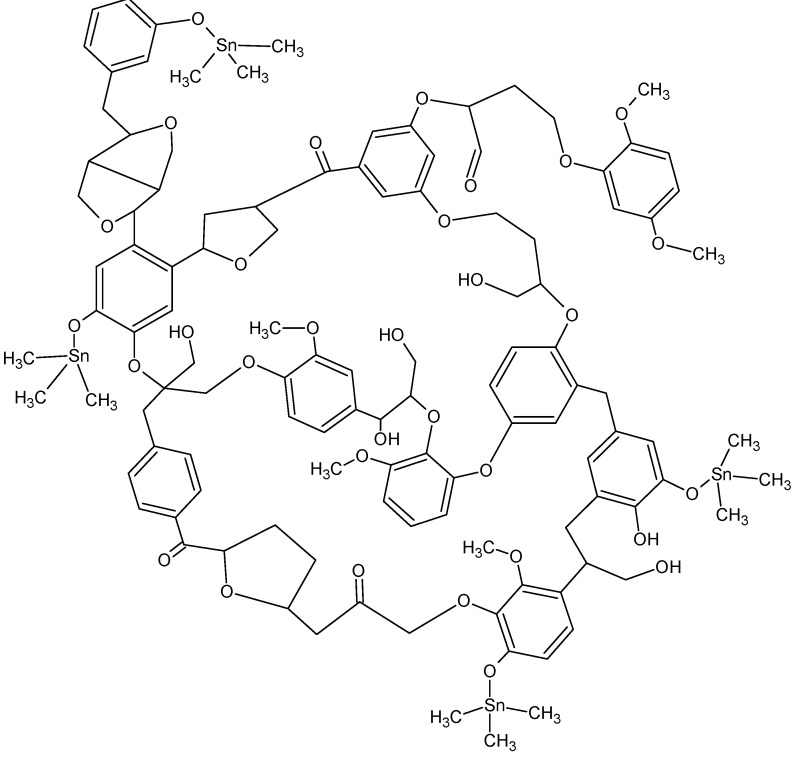
Representative structure of lignin modified through reaction with trimethyltin chloride.

The actual structure of lignin, while in reality three dimensional, is often treated as two dimensional because much of the structure is sandwiched between walls within the plant giving it a small molecular thickness. Figure 12 contains a representative structure of lignin and Figure 13 shows a representative structure of a lignin product from reaction with trimethyltin chloride. 

We have synthesized a wide variety of lignin-containing products from reaction of organotin mono- and dihalides with lignin [2,127]. Lignin has both aliphatic and aromatic hydroxyl groups. The pKa for the aromatic hydroxyl groups is in the range of 10 while the pKa for the aliphatic hydroxyls is about 14. Thus, under the reaction conditions employing sodium hydroxide as the base the aromatic hydroxyl group will be deprotonated making it a stronger nucleophile than the protonated aliphatic hydroxyl. Counter, the reaction occurs within the organic phase so that the deprotonated group will be less likely to be within the reaction zone. Mass spectrometry shows a preponderance of AR-O-Sn compared with R-O-Sn units consistent with the deprotonation being more important in the generation of reaction sites for reaction with the organotin halide. For structural considerations empirical C_9_ units are typically used where there are variable numbers of hydroxyls present in this C_9_ unit. The lignin employed for our tests has 0.31 hydroxyls per C_9_ unit. From reaction with a variety of mono, di, and tri-alkyl and aryltin halides the percentage hydroxyl groups that contain organotin units vary from about 20% to 70%. As solids, these products show almost no inhibition of a number of bacteria but some do exhibit good inhibition of *C. albicans* [128].

In another study, organotin halides were added to mixtures of lignin and dihydroxyl and monohydroxyl-terminated poly(ethylene glycols) [2,129]. The employed PEG had about 45 ethylene glycol units between the terminal hydroxyl groups and the employed monohydroxyl-terminated material had about 35 ethylene glycol units in it. The system was constructed so that the ethylene glycol chains connected the lignin units with the connecting groups being derived from the organostannane dihalides. These products had greater dimensional stability compared to the lignin product alone. The products could be made into flexible films. These materials also exhibited selective inhibition of *C. albicans* [129]. 

#### 3.1.3. Poly(vinyl alcohol)

Poly(vinyl alcohol), PVA, is produced by the hydrolysis of poly(vinyl acetate), PVAc. Because of the ability to hydrogen bond and small size of the hydroxyl grouping, PVA is a crystalline atactic material. Since PVA forms strong internal hydrogen bonds, completely hydrolyzed PVAc is not water soluble. Thus, hydrolysis of PVAc is stopped giving a material whose hydroxyl content can be varied but for most commercial uses it is about 88% hydrolyzed and water soluble. The PVA employed by us was 88% hydrolyzed. This means that the maximum loading or substitution on PVA is limited by the extent of hydrolysis of the original PVAc and steric restrictions. 

**Scheme 3:**
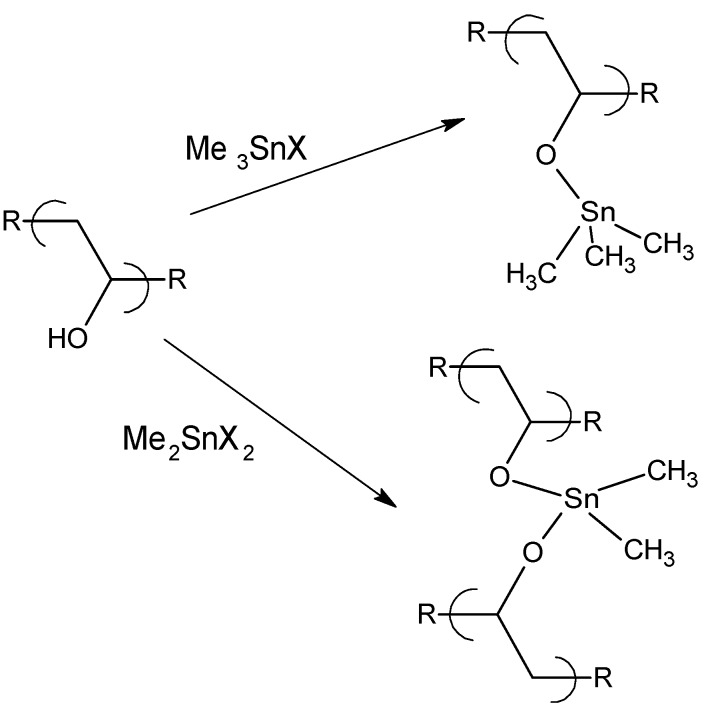
Products from reaction of poly(vinyl alcohol) with organotin mono and dihalides.

Products from dihaloorganotin are cross-linked while those from monohaloorganotin reactants are linear and soluble as illustrated in Scheme 3 for products from dimethyltin and trimethyltin [130]. Flexible films and fibers can be made from the linear products. The extent of inclusion of the organotin moiety in PVA can be controlled through use of different ratios of reactants. At high molar concentrations of PVA compared to the organotin reactant, the amount of organotin moiety within the product is less in comparison to employing high ratios of the organotin reactant where the amount of organotin is higher. Inhibition of various microorganisms is largely dependent on the nature of the organotin moiety. For instance, the trimethyltin products show no inhibition of bacteria such as *E. coli*, *P. Auer*., and *S. aureus* but they do inhibit *C. albicans*. The tripropyltin and triethyltin products generally exhibit good inhibition of all of the tested microorganisms. As in the case with lignin, all of the products show outstanding inhibition of *C. albicans*. 

The products from PVA and some from lignin deserve special mention because of their ability to inhibit *Candia albicans*, the microorganism most responsible for yeast infections in women and men [131,132]. For many of the organotin-containing polymers this inhibition is selective leaving much of the natural flora intact. The PVA products were compared to commercial preparations and dramatically out performed them. In general the commercial preparations required repeated treatments to completely inhibit the *C. albicans* whereas the PVA products were able to inhibit *C. albicans* in one treatment [131,132,133]. 

### 3.2. Synthesis of Polysaccharides

Carbohydrates are the most abundant organic compounds, constituting three-fourths of the dry weight of the plant world. They represent a great storehouse of energy as a food for humans and animals. About 400 billion tons of sugars are produced annually through photosynthesis, dwarfing the production of other natural polymers, with the exception of lignin. Much of this synthesis occurs in the oceans, pointing to the importance of harnessing this untapped food, energy, and renewable feedstocks storehouse. 

The potential complexity of even the simple aldohexose monosaccharides is indicated by the presence of five different chiral centers, giving rise to 2^5^ or 32 possible steroisomeric forms of the basic structure, two of which are glucose and mannose. While these sugars differ in specific biological activity, their gross chemical reactivities are almost identical, permitting one to often employ mixtures within chemical reactions without regard to actual structure. Their physical properties are also almost the same, again allowing for the mixing of these structures with little change or loss in physical behavior. But their biological behavior may vary greatly. 

We have synthesized a wide variety of products derived from saccharides and complex saccharides mainly from sucrose, dextran, cellulose, and xylan [86,88,134,135,136,137,138,139,140,141,142,143,144]. This has been reviewed [2]. 

**Figure 14:**
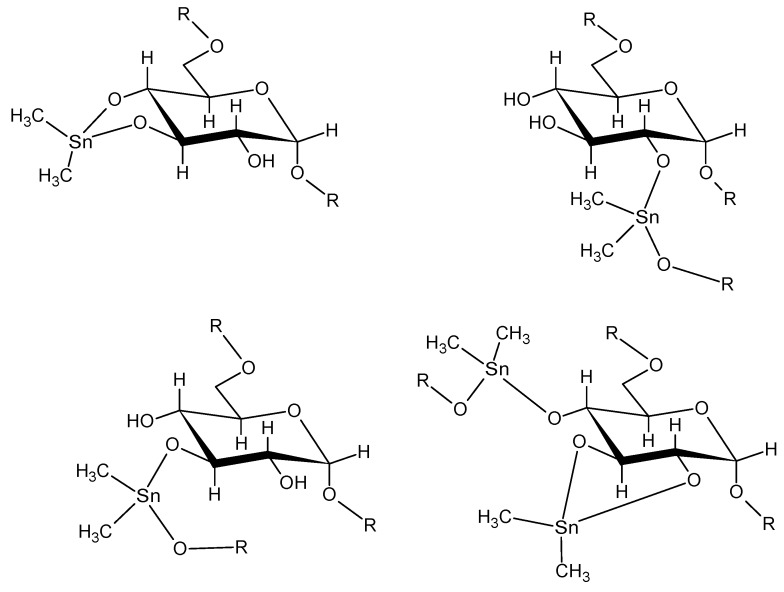
Structural units present in dextran modified through reaction with dimethyltin dichloride.

Carbohydrates are diverse with respect to occurrence and size. For illustrative purposes for complex carbohydrates we will use dextran. Dextran is an extracellular polysaccharide synthesized by certain bacteria when grown on sucrose. The polymer consists mainly of branched chains of alpha(1->6)-linked D-glucosepyranose units. Reaction with organotin dihalides (here dimethyltin dihalide) results in a complex of units including unreacted units, those where the organotin forms preferred five-member rings such as shown below upper left, units where one organotin moiety is bonded to another glucosepyranose unit as shown in the upper right and lower left Figure 14, or more than one organotin moiety can be associated with a single glucosepyranose unit, lower right (Figure 14). 

**Figure 15:**
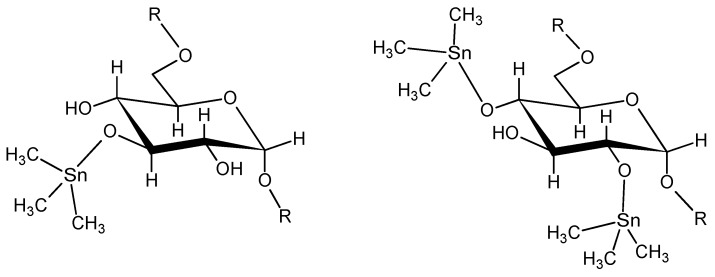
Representative units in dextran for reaction with trimethyltin chloride.

Reaction with monohalo organotins (here trimethyltin halide) again gives a mixture of products, but it does not increase the crosslinking so that these products are soluble. The mixture contains unreacted units, units containing one organotin moiety (left, below; Figure 15), and units containing more than one organotin moiety per unit (right, below; Figure 15). 

**Figure 16:**
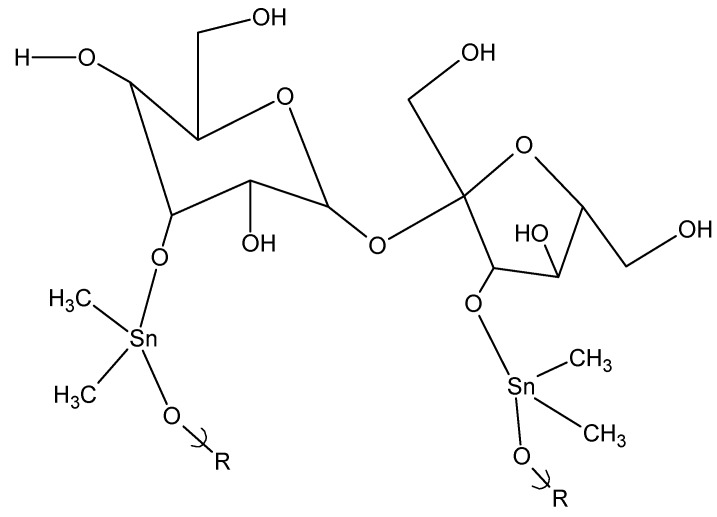
Structural units present in the product from reaction of sucrose and organotin dihalides.

For simplicity, we will also briefly describe our efforts with the simple sugar sucrose. As expected these products are cross-linked because we employed dialkyltin and diaryltin dihalides as reactants with the primary sites of reaction being the ring-hydroxyls as shown in Figure 16 for a dimethyltin-containing moiety.

## 4. Conclusions

The organotin polyethers offer a number of potentials within the medical arena. Some show a good ability to inhibit the growth of a wide variety of generally unwanted materials including bacteria, fungus, cancer, and viruses. But these are potentials and await actuality.

Several things are important to remember concerning the organotin polyethers described in this review. First, all are synthesized from commercially available monomers. Second, synthesis is generally rapid occurring within 30 second or less under room conditions of pressure and temperature. Third, synthesis generally is affected employing the interfacial condensation technique that is currently employed for the synthesis of important commercial polymers, namely polycarbonates and aramids. Thus, scale up should be straightforward producing mg to tons of product. 
